# Diminished liver microperfusion in Fontan patients: A biexponential DWI study

**DOI:** 10.1371/journal.pone.0173149

**Published:** 2017-03-03

**Authors:** Hildebrand Dijkstra, Djoeke Wolff, Joost P. van Melle, Beatrijs Bartelds, Tineke P. Willems, Matthijs Oudkerk, Hans Hillege, Aad P. van den Berg, Tjark Ebels, Rolf M. F. Berger, Paul E. Sijens

**Affiliations:** 1 Center for Medical Imaging—North East Netherlands, Department of Radiology, University Medical Center Groningen, University of Groningen, Groningen, The Netherlands; 2 Center for Congenital Heart Diseases, Department of Pediatric Cardiology/Beatrix Children's Hospital, University Medical Center Groningen, University of Groningen, Groningen, The Netherlands; 3 Center for Congenital Heart Diseases, Department of Cardiology, University Medical Center Groningen, University of Groningen, Groningen, The Netherlands; 4 Department of Radiology, University Medical Center Groningen, University of Groningen, Groningen, The Netherlands; 5 Center for Medical Imaging—North East Netherlands, University Medical Center Groningen, University of Groningen, Groningen, The Netherlands; 6 Department of Epidemiology, University Medical Center Groningen, University of Groningen, Groningen, The Netherlands; 7 Department of Gastroenterology, University Medical Center Groningen, University of Groningen, Groningen, The Netherlands; 8 Center for Congenital Heart Diseases, Department of Cardiothoracic Surgery University Medical Center Groningen, University of Groningen, Groningen, The Netherlands; Texas Technical University Health Sciences Center, UNITED STATES

## Abstract

It has been demonstrated that hepatic apparent diffusion coefficients (ADC) are decreasing in patients with a Fontan circulation. It remains however unclear whether this is a true decrease of molecular diffusion, or rather reflects decreased microperfusion due to decreased portal blood flow. The purpose of this study was therefore to differentiate diffusion and microperfusion using intravoxel incoherent motion (IVIM) modeled diffusion-weighted imaging (DWI) for different liver segments in patients with a Fontan circulation, compare to a control group, and relate with liver function, chronic hepatic congestion and hepatic disease. For that purpose, livers of 59 consecutively included patients with Fontan circulation (29 men; mean-age, 19.1 years) were examined (Oct 2012─Dec 2013) with 1.5T MRI and DWI (b = 0,50,100,250,500,750,1500,1750 s/mm^2^). IVIM (D_slow_, D_fast_, f_fast_) and ADC were calculated for eight liver segments, compared to a control group (19 volunteers; 10 men; mean-age, 32.9 years), and correlated to follow-up duration, clinical variables, and laboratory measurements associated with liver function. The results demonstrated that microperfusion was reduced (p<0.001) in Fontan livers compared to controls with ─38.1% for D_fast_ and ─32.6% for f_fast_. Molecular diffusion (D_slow_) was similar between patients and controls, while ADC was significantly lower (─14.3%) in patients (p<0.001). ADC decreased significantly with follow-up duration after Fontan operation (r = ─0.657). D_slow_ showed significant inverse correlations (r = ─0.591) with follow-up duration whereas D_fast_ and f_fast_ did not. From these results it was concluded that the decreasing ADC values in Fontan livers compared with controls reflect decreases in hepatic microperfusion rather than any change in molecular diffusion. However, with the time elapsed since the Fontan operation molecular diffusion and ADC decreased while microperfusion remained stable. This indicates that after Fontan operation initial blood flow effects on the liver are followed by intracellular changes preceding the formation of fibrosis and cirrhosis.

## Introduction

Diffusion-weighted imaging (DWI) has been successfully applied in the assessment of diffuse liver diseases such as cirrhosis, fibrosis and steatosis [1–6]. Cirrhotic livers had significantly lower apparent diffusion coefficients (ADC) than normal livers [2,3,6] and negative correlations between fibroses stages and ADC values were demonstrated [1,4,5]. The ADC is obtained by calculating a mono-exponential fit from multiple (al least two) diffusion-weighted images, thereby integrating molecular diffusion and microperfusion effects in one quantitative parameter [7,8]. The concept of the ADC however has been derived from the more complex intravoxel incoherent motion (IVIM) model, which separates molecular diffusion and microperfusion effects by fitting a bi-exponential model to multiple DW images [8]. It has been suggested that the ADC reduction observed in cirrhotic livers could be linked to decreased microperfusion values and may be related to reduced perfusion [2].

A category of patients with altered hepatic perfusion are patients with a Fontan circulation. Fontan et al. described a palliative operation in which the right atrium (and in newer techniques the caval veins) is directly connected to the pulmonary arteries [9,10]. Additional detail about the Fontan operation is provided in S1 Appendix. In the absence of a subpulmonary ventricle, this operation induces increased central venous pressure, decreased preload and increased afterload of the ventricle [11]. In the Netherlands, yearly around 1200 newborns are born with a congenital heart disease and around 4–5% of these patients have a complex congenital heart disease, known as the univentricular heart, and can be subject for a Fontan operation [12–14]. Over four decades, the short term survival after the Fontan operation improved significantly, resulting in an increasing cohort of Fontan patients who reach adolescence and adulthood [15]. Consequently, long-term complications of the Fontan circulation are more commonly seen.

One of the implications of the Fontan circulation is liver disease resulting in fibrosis and cirrhosis [16–19]. A significant positive correlation has been found between the follow-up duration (number of days since the Fontan operation) and the degree of hepatic fibrosis [20]. This hepatic damage in the context of a Fontan circulation is presumably caused by the elevated venous pressure and limited cardiac output that causes decreased portal flow [14]. The hepatic artery compensates the diminished portal flow by increased hepatic arterial flow, which is termed the hepatic arterial buffer response. The distribution of the microperfusion is likely to vary among the different liver segments due to the alternative distribution of the hepatic flow in Fontan patients.

In a recent report we showed that mean hepatic ADCs are decreased in Fontan patients [21]. It remained unclear whether this is a true decrease of molecular diffusion, or rather reflects decreased microperfusion due to decreased portal blood flow. Therefore, the aim of our current analysis is to differentiate diffusion and microperfusion using IVIM modeled DWI for different liver segments in patients with a Fontan circulation, compare the results to a control group, and explore the relationship with follow-up duration, liver function, chronic hepatic congestion and hepatic disease.

## Materials and methods

### Ethics statement

The protocol and consent procedure of the study was approved by the Medical Ethics Review Board of the University Medical Center Groningen, and written informed consent was obtained for each patient. For children and minors written informed consent was obtained from the parents or and/or their legally authorized representative. All participants written consent forms with date and signature were archived and the study was conducted in accordance with the ICH-GCP declaration of Helsinki.

### Study population

Between January 2012 and October 2013, consecutive patients with a functionally univentricular heart treated with a Fontan operation (further referred to as Fontan patients) were scheduled for cardiac MRI including diffusion-weighted imaging (DWI) of the liver [21]. Inclusion criteria were: age 10 years or older. This resulted in 59 patients, 32 children and 27 adults (29 men; mean-age, 19.1 years; age-range, 9.6–44.7 years). All 59 patients have been previously reported [21]. This prior article dealt with the association between the ADC and functional liver parameters; whereas in this manuscript we apply IVIM modeling to explain the previously observed decreased ADC in Fontan livers by measuring the microperfusion and molecular diffusion in each of eight liver segments.

Clinical variables were available and included body mass index (BMI), cardiac index, ejection fraction, end-diastolic volume (EDV), laboratory measurements (AST, ALT, γ-GT, FactorVIII, AST/ALTratio, bilirubin, albumin, PT), MELDXI (model for end-stage liverdisease excluding INR), Fib-4 (Fibrosis-4 score) and vena cava inferior (VCI) diameter and were obtained using previously described standardized methods [21].

In addition, a control group of 19 volunteers was included in this study: 10 men and 9 women (mean-age, 32.9 years; age-range, 20–62 years) [22]. All volunteers had no relevant medical history.

### MR protocols

Diffusion-weighted imaging (DWI) of the liver was acquired by Magnetic Resonance Imaging (MRI), using a commercially available 1.5 T scanner (Magnetom Aera, Siemens Medical Solutions, Erlangen, Germany). A 32 element spine matrix coil in combination with a 4 element body matrix was used as the receiver, and the body coil as transmitter. The protocol included a routine localizer where after 9 series (b = 0, 50, 100, 250, 500, 750, 1000, 1500, 1750 s/mm^2^) of DWI were acquired using a spin echo based echo-planar imaging (EPI) sequence using the following parameters: TR 5900–9600 ms; TE 90 ms; slice-thickness 5 mm; slice gap 10 mm; FOV 242×300 mm^2^; matrix 116×144; bandwidth 1335 Hz/pixel; averages 4 and parallel acquisition technique GRAPPA with acceleration factor 2. PACE respiratory triggering was enabled and spectral adiabatic inversion recovery (SPAIR) was used for fat suppression to avoid artifacts from subcutaneous fat. In total, between 14 and 16 transverse slices were acquired to cover the whole liver within an acquisition time of 2.5 minutes.

### DWI analysis

The control group was acquired using 7 b-values (b = 0, 50, 100, 250, 500, 750, 1000 s/mm^2^); therefore only these 7 b-values were used in the comparison between Fontan patients and controls, whereas the remaining acquired DWI series (b = 1500 and 1750 s/mm^2^) were included in all other analyses.

Drawing of regions-of-interest (ROIs) and the analysis were performed off-line using monoexponential (ADC) and biexponential fitting procedures in a programmable graphical and calculus environment (Matlab, The Mathworks, Natick, MA, USA). Circular ROIs of 21.5 mm^2^ were drawn in 8 different segments of the liver (segment II, III, IVa, IVb, V, VI, VII, VIII) according to the Couinaud-Bismuth classification [23,24]. Extra care was taken to avoid major blood vessels in the ROIs.

In the biexponential analyses, the diffusion-weighted signal intensities S were fitted using the parameters prescribed by the IVIM model [8,25]:

$$\frac{S}{S_{0}}=f_{fast}\cdot exp(-b\cdot D_{fast})+f_{slow}\cdot exp(-b\cdot D_{slow})$$ (1)

where S_0_ is the maximum signal intensity, D_fast_ is the fast component representing microperfusion, f_fast_ is the fraction of microperfusion, D_slow_ is the slow component representing molecular diffusion and f_slow_ is the fraction of molecular diffusion (f_slow_ = 1—f_fast_). Eq 1 was fitted by the Nelder-Mead simplex direct search method with bound constraints, which performs a constrained non-linear minimization of the sum of the squared residuals [26,27]. The initial guess D^0^_slow_ was estimated by calculating the slope of the asymptote by monoexponential fitting of the slow signal component between b = 500 and 1000 s/mm^2^, and D_slow_ was bound between 0.2 and 5 × D^0^_slow_ × 10^−3^ mm^2^/s. The intercept of the asymptote with the y-axis at S_0_ resulted in an initial guess f^0^_fast_, and f_fast_ was bound between 0 and 1. The slope of the signal between b = 0 and b = 50 s/mm^2^ was used to guess the initial value of the fast signal component (D^0^_fast_), and D_fast_ was bound between D^0^_slow_ (microperfusion can never be slower than molecular diffusion) and 100 × 10^−3^ mm^2^/s. The ADC was obtained by using a clinically accepted method: a mono-exponential fit of all b-values was performed.

### Statistics

Statistical analyses were performed using SPSS (SPSS 20, Chicago, IL, USA). All data were tested for normality using Shapiro–Wilk tests. IVIM parameters and ADC averaged over all liver segments were compared between Fontan patients and controls by independent samples t-tests. Subsequently, IVIM and ADC were compared per liver segment between Fontan patients and controls by independent samples t-tests. One-way ANOVA tests were used to compare IVIM parameters and ADC between the eight liver segments.

Correlations between DWI (D_slow_, D_fast_, f_fast_ and ADC) and clinical laboratory measurements and follow-up duration were calculated using a linear (Y = a∙X + b) model using Pearson’s correlation coefficient for normally distributed variables and Spearman's rank correlation coefficient for non-normally distributed variables.

Normally distributed data were shown as means with standard deviations. Non-normally distributed data were shown as medians with interquartile range. For all statistical tests P < 0.05 was considered to indicate a statistically significant difference.

## Results

IVIM-DWI, ADC, AST, ALT, γ-GT, FactorVIII, AST/ALTratio, EDV, EF and Cardiac index were normally distributed (p ≥ 0.071). Microperfusion parameters (D_fast_ and f_fast_) averaged over all segments were significantly lower in Fontan patients compared to controls. D_fast_ was 23.2 × 10^−3^ mm^2^/s in the liver of Fontan patients, and 37.5 × 10^−3^ mm^2^/s in controls (p<0.001, ─38.1%). F_fast_ was 23.6% in Fontan patients, and 35.0% in controls (p<0.001, ─32.6%). D_slow_ was similar in patients and controls ranging between 0.95 × 10^−3^ mm^2^/s and 1.00 × 10^−3^ mm^2^/s (p = 0.171). The ADC was significantly lower (p<0.001, ─14.3%) in Fontan patients (1.08 × 10^−3^ mm^2^/s) compared to the controls (1.26 × 10^−3^ mm^2^/s).

Also on a segmental level, the microperfusion parameters were significantly decreased for the majority of liver segments of Fontan patients compared to controls (Table 1). The molecular diffusion was significantly lower in half of the segments (III, IVb, VI and VII) compared to controls (Table 2). The ADC was significantly lower in almost all segments (except segment V).

**Table 1:** Microperfusion data (using 7 b-values) per segment.

	D_fast_ (×10^−3^ mm^2^/s)				F_fast_ (%)			
Seg.	Controls	Patients	Δ%	P	Controls	Patients	Δ%	P
II	21.5 ± 8.8	27.0 ± 14.1	+25.6	0.051	58.6 ± 12.1	32.6 ± 12.2	-79.8	<0.001*
III	37.7 ± 22.2	24.6 ± 8.9	-53.3	0.021*	37.9 ± 11.0	24.9 ± 9.8	-52.2	<0.001*
IVa	31.2 ± 17.9	23.8 ± 9.5	-30.5	0.095	39.4 ± 16.9	25.1 ± 10.4	-57.0	0.002*
IVb	46.3 ± 16.9	22.9 ± 8.8	-202	<0.001*	35.1 ± 9.5	21.2 ± 7.9	-65.6	<0.001*
V	37.5 ± 13.2	22.2 ± 8.0	-68.9	<0.001*	29.3 ± 6.9	20.4 ± 8.3	-43.6	<0.001*
VI	45.1 ± 23.7	22.4 ± 8.1	-201	0.001*	27.6 ± 9.2	21.3 ± 7.9	-29.6	0.012*
VII	42.9 ± 25.1	21.4 ± 8.9	-200	0.002*	27.1 ± 7.1	21.7 ±6.4	-24.9	0.007*
VIII	37.8 ± 15.2	22.0 ± 8.6	-71.8	<0.001*	24.9 ± 9.7	22.2 ± 7.0	-12.2	0.293
P	0.045*	0.001*			<0.001*	<0.001*		

Differences among the segments were tested using one-way ANOVA tests. Differences of DWI data between patients and controls were assessed by independent t-tests. the microperfusion parameters were significantly decreased for the majority of liver segments of Fontan patients compared to controls. Data are mean ± standard deviations.

* P-value indicates significant difference.

**Table 2:** Diffusion data (using 7 b-values) per segment.

	ADC (×10^−3^ mm^2^/s)				D_slow_ (×10^−3^ mm^2^/s)			
Seg.	Controls	Patients	Δ%	P	Controls	Patients	Δ%	P
II	1.42 ± 0.29	1.15 ± 0.28	-23.5	<0.001*	0.79 ± 0.40	0.95 ± 0.36	+16.8	0.123
III	1.38 ± 0.15	1.10 ± 0.15	-25.5	<0.001*	1.12 ± 0.21	0.96 ± 0.19	-16.7	0.009*
IVa	1.40 ± 0.24	1.12 ± 0.16	-25.0	<0.001*	1.05 ± 0.38	0.97 ± 0.23	-8.2	0.422
IVb	1.32 ± 0.15	1.09 ± 0.11	-21.1	<0.001*	1.12 ± 0.14	0.98 ± 0.14	-14.3	0.001*
V	1.09 ± 0.18	1.05 ± 0.12	-3.8	0.391	0.92 ± 0.21	0.96 ± 0.17	+4.2	0.482
VI	1.18 ± 0.08	1.06 ± 0.15	-11.3	<0.001*	1.02 ± 0.09	0.95 ± 0.17	-7.4	0.021*
VII	1.21 ± 0.10	1.06 ± 0.14	-14.2	<0.001*	1.05 ± 0.13	0.93 ± 0.18	-12.9	0.005*
VIII	1.09 ± 0.16	1.00 ± 0.15	-9.0	0.024*	0.94 ± 0.21	0.88 ± 0.20	-6.8	0.213
P	<0.001*	<0.001*			0.001*	0.208		

Differences among the segments were tested using one-way ANOVA tests. Differences of DWI data between patients and controls were assessed by independent samples t-tests. The molecular diffusion was significantly lower in half of the segments compared to controls. The ADC of Fontan patients was significantly lower in almost all segments compared to controls (except segment V). Data are mean ± standard deviations.

* P-value indicates significant difference.

Concerning the homogeneity of IVIM values among the segments, it was observed that for Fontan patients the microperfusion parameters differed significantly throughout the liver (p ≤ 0.045). This was also true for the ADC (p<0.001). The molecular diffusion however was similar among the segments (p = 0.208).

**Fig 1:**
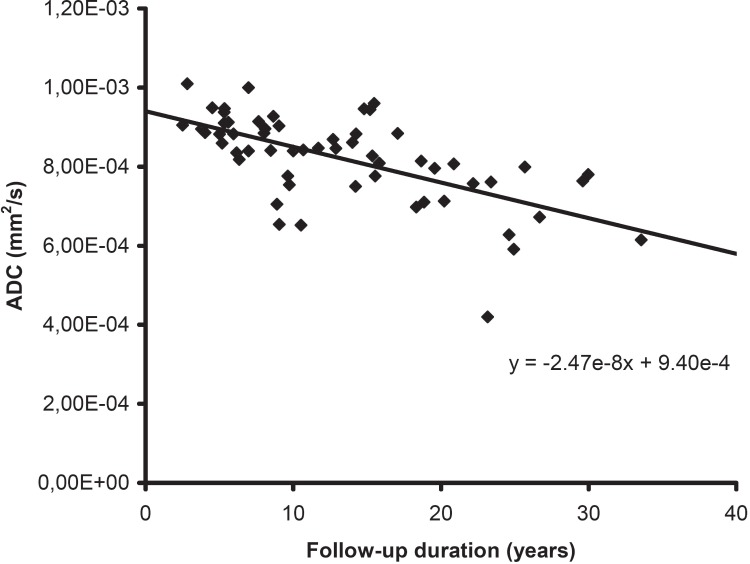
Correlation between follow-up duration and apparent diffusion coefficient (ADC). The mean hepatic ADC for each patient (n = 59) is plotted against the number of years since the Fontan operation (follow-up duration). The ADC showed a significant negative linear relationship with the follow-up duration (r = ─0.657).

**Fig 2:**
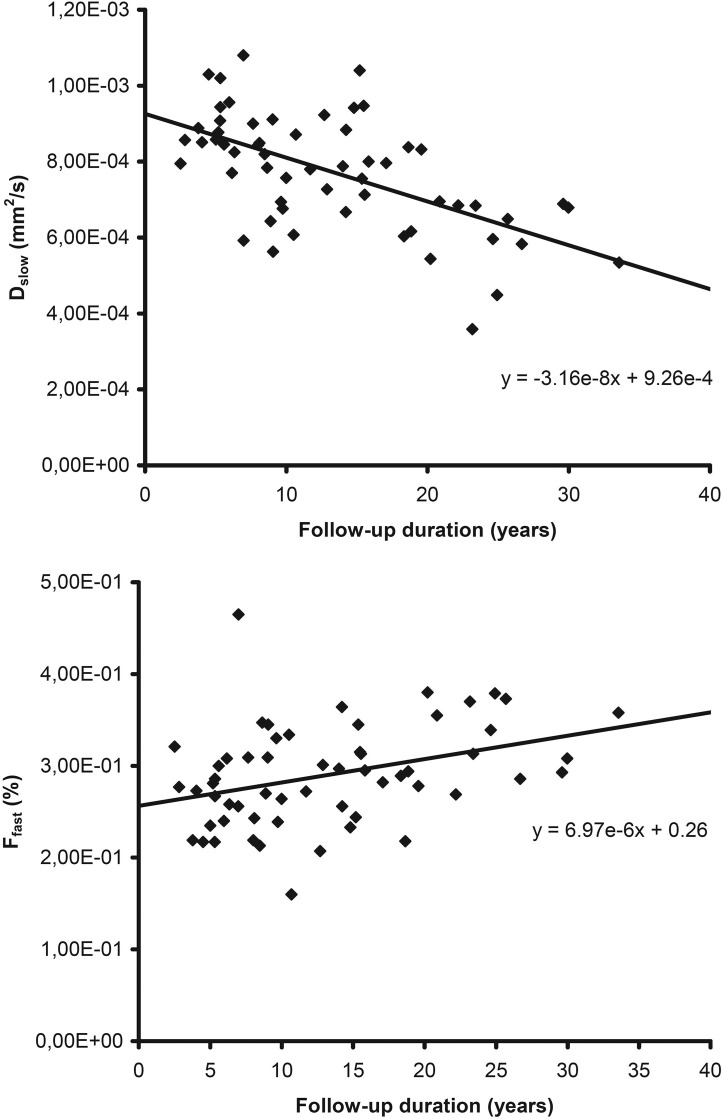
Correlation between follow-up duration and IVIM-DWI. For each patient (n = 59) the mean hepatic molecular diffusion (D_slow_) and fraction of microperfusion (f_fast_) are plotted against the number of years since the Fontan operation (follow-up duration). The molecular diffusion (top) showed a significant negative linear relationship (r = ─0.591) with the follow-up duration. The fraction of microperfusion (bottom) showed a significant positive linear relationship (r = +0.401) with the follow-up duration.

The DWI data averaged over all segments were correlated to the clinical laboratory measurements (Table 3). The median follow-up time was 11.2 years (min: 2.5 years; max: 33.6 years). The ADC showed a significant negative linear relationship with the follow-up duration after Fontan operation with a correlation coefficient r = ─0.657 (Fig 1), with the highest correlations found in segments II and VIII (Table 4). Also the molecular diffusion showed a significant negative linear relationship (r = ─0.591) with the follow-up duration (Fig 2), with the highest correlations found in segments V and VIII. The microperfusion was stable over time and did not correlate with the follow-up duration (r = ─0.158). The fraction of microperfusion on the other hand showed a significant positive linear relationship (r = +0.401) with the follow-up duration (Fig 2), with the highest correlations in segments V and VIII.

**Table 3:** Correlations between DWI parameters and clinical variables.

	ADC	D_slow_	D_fast_	F_fast_
**Laboratory measurements**				
AST†	+0.199	+0.275*	+0.250	-0.132
ALT†	-0.173	-0.188	+0.045	+0.218
gamma GT†	-0.450*	-0.424*	-0.047	+0.199
Bilirubin‡	-0.258	-0.275	+0.198	+0.301*
Albumin‡	+0.127	+0.100	+0.238	+0.110
PT‡	-0.143	-0.180	-0.321*	-0.033
Factor VIII†	+0.046	-0.003	-0.058	+0.005
**Liver disease scores**				
MELDXI‡	-0.259	-0.271	+0.266	+0.402*
AST-ALT ratio†	+0.330*	+0.405*	+0.203	-0.317*
Fib-4‡	-0.344*	-0.322*	-0.020	+0.324*
**Cardiac function**				
EDV†	+0.153	+0.093	+0.031	+0.131
EF†	+0.043	+0.076	+0.070	-0.106
Cardiac-index†	+0.270*	+0.266*	+0.220	+0.005
VCI diameter ‡	-0.222	-0.211	0.034	0.180
Follow-up duration ‡	-0.657*	-0.591*	-0.158	+0.401*

† Pearson’s correlation coefficient.

‡ Spearman's rank correlation coefficient.

* P-value indicates significant difference.

**Table 4:** Follow-up duration and DWI parameters correlated per segment.

Segment	ADC	D_slow_	D_fast_	F_fast_
II	-0.632*	-0.307*	+0.002	-0.167
III	-0.447*	-0.397*	+0.067	+0.239
IVa	-0.555*	-0.453*	-0.216	+0.212
IVb	-0.367*	-0.258	+0.013	+0.221
V	-0.562*	-0.556*	-0.120	+0.440*
VI	-0.494*	-0.488*	-0.123	+0.269*
VII	-0.328*	-0.367*	-0.207	+0.203
VIII	-0.612*	-0.567*	-0.154	+0.371*

Data are Spearman's rank correlation coefficients.

* P-value indicates significant difference.

The FIB-4 score showed weak though significant relationships, negative with molecular diffusion (r = ─0.322) and positive with the fraction of microperfusion (r = +0.324). Some other clinical laboratory parameters also showed significant correlations with IVIM-DWI parameters, most notably gamma GT with ADC and D_slow_ (r = ─0.450 and r = ─0.424, respectively; Table 3).

## Discussion

This study demonstrates that decreased hepatic ADC measurements of Fontan patients can be explained by significantly lower microperfusion in the Fontan liver rather than by decreased diffusion. It was observed that the molecular diffusion (D_slow_) was similar between Fontan patients and controls, while the microperfusion parameters (D_fast_ and f_fast_) and ADC were significantly lower in the Fontan liver.

A previously formulated hypothesis relating hypoperfusion of the liver to the reduced ADC in Fontan patients is thus substantiated [21]. However, the currently and previously [21] reported strong negative dependency of the hepatic ADC on the follow-up duration after the Fontan operation, reflects changes of the molecular diffusion with time rather than further changes in microperfusion. This indicates that initial blood vessel effects leading to decreases in microperfusion and hepatic congestion, are followed by true cellular changes leading to fibrosis and cirrhosis.

The evidence in the current study that hypoperfusion of the liver in Fontan patients causes the reduced ADC values as compared with controls, confirms the high degree of sensitivity to microperfusion of the mono-exponential model which was already shown decades ago by Le Bihan et al. in DWI of the brain [8]. When the DWI sequence contains b-values in the microperfusion range (b ≤ 100 s/mm^2^), and the microperfusion is diminished, the ADC measurements will decrease [22].

With a bi-exponential IVIM model, the cellular diffusion component can be distinguished from the microperfusion component. This improves our understanding of the underlying pathophysiology of liver disease in the Fontan circulation by providing important additional information on the association between hepatic hypoperfusion and hepatic congestion and subsequent cellular changes leading to decreases in molecular diffusion, with the formation of liver fibrosis and cirrhosis in clinical practice.

It was observed that the ADC values and molecular diffusion decreased with the follow-up duration after Fontan operation, whereas the microperfusion was stable over time. In other words, structural liver disease (i.e. liver fibrosis or cirrhosis) seems to be preceded by reduced cellular diffusion, not present at first but developing progressively in time after Fontan operation.

The relationship between heart failure and liver dysfunction was first described in detail by Sherlock in 1951 [28]. Hepatic complications of heart failure comprise a spectrum of combined cardiac and hepatic disorders [Naschirtz2000]. It is considered that both hepatic congestion and low perfusion of the liver are the causative mechanisms of liver complications [29,30]. Both conditions can occur simultaneously, however there are no studies describing any causality between hepatic hypoperfusion and increasing degrees of hepatic congestion.

There are several other relations however demonstrated up to now. Katzkin et al. showed that the number of patients with fibrosis is related to the duration of hepatic congestion [Katzkin1939]. A number of other studies demonstrated that diminished liver perfusion causes increasing degrees of fibrosis and cirrhosis [31–34]. Also decreasing ADCs have been demonstrated with each fibrosis stage, confirmed by histopathology [1,4,5]. So these studies suggest that hepatic congestion and hypoperfusion of the liver both lead to fibrosis and cirrhosis.

The patients in the current showed abnormal values associated with hepatic congestion (elevated γ-GT or alkaline phosphatase) [21]. Both chronic venous congestion and hepatic hypoperfusion due to restricted cardiac output is therefore suggested as potential mechanism leading to structural liver damage in Fontan patients. Considering that the hepatic perfusion in this study did not correlate with the follow-up time since the Fontan operation, this suggests that the hepatic hypoperfusion and congestion are chronic and stable over time.

All patients had some derangement of laboratory liver measurements; potentially laboratory disturbance is not only associated with advanced liver disease, but also influenced by chronic liver damage, due to congestion and hypoperfusion. Increased gamma GT, a sign of congestive hepatopathy, was related to D_slow_ and unrelated to the microperfusion. This gives further confirmation that D_slow_ is related to liver fibrosis and cirrhosis, and suggests that in the liver these processes might develop faster in context of more liver congestion (as in the first case report by Lemmer in 1983) [35].

Previous histological studies have demonstrated, on a microscopic level, that in patients with chronic hepatic congestion, the poorly arterially supplied hepatocytes in the centrilobular zone show atrophy [36,37]. In patients with a Fontan circulation, atrophy of centrilobular hepatocytes seems related to the degree of right sided pressure and to the time after Fontan operation [20,37]. Likewise, on a macroscopic level, the arterial blood supply is not homogenously distributed over the various liver segments. It has been reported that the ratio of the arterial liver perfusion (ALP) and portal venous perfusion (PVP) varies and is the lowest in segments V to VIII and highest in segments I to IV [38,39]. When the ALP over-compensates the PVP in Fontan patients, it is expected that the microperfusion increases in segments I to IV, and diminishes in segments V to VIII. This is confirmed by our data. This suggests that, in a Fontan circulation, the development of liver fibrosis or cirrhosis varies between the different liver segments, depending on the degree of arterial blood supply.

Altogether, this study provides strong evidence that the degree of congestion as reflected in the hepatic perfusion is generally stable with time after Fontan operation, whereas the subsequent hepatocellular diffusion decreases are associated with liver fibrosis/-cirrhosis development. With the bi-exponential model, the DWI-MR technique provides the opportunity to distinguish between these two components. For clinical practice, this provides a major advantage compared to the other non-invasive alternatives for liver biopsy. Potentially, a decrease in the microperfusion component could indicate an adverse change in the Fontan circulation, for instance more congestion due to a conduit stenosis or pulmonary vascular remodeling. With a routine follow-up of the cellular diffusion, the development of liver fibrosis/-cirrhosis can be safely monitored. We suggest further research to investigate changes in microperfusion and cellular diffusion longitudinally, and want to highlight that, with progressive liver disease being apparently inherent to the Fontan circulation, steps have to be taken concerning potential treatment options for liver disease in Fontan patients. Therefore, future studies should focus on reversibility of this liver disease, and the effects and timing of potential treatment options, including heart transplantation, Fontan conversion or a late Fontan takedown.

### Limitations

Liver damage in the Fontan circulation presents with disturbed transaminases, coagulation disorders, and can eventually lead to liver fibrosis-, cirrhosis and even hepatocellular carcinoma [40–42]. It was assumed that the severity of fibrosis increases with the follow-up duration after Fontan operation. The stage of liver fibrosis or cirrhosis was however not confirmed by liver biopsy, thereby limiting the study.

Although the segmental differences in microperfusion strongly point to the reported variation in the ALP and PVP in Fontan patients, this might also be related to cardiac pulsation artifacts in DWI which are known to result in deviating values between right and left liver lobe. The increase in the ADC in the left lobe is usually explained from the increased cardiac motion in the left lobe [43–47]. However, it was also demonstrated that the increased ADC in the left lobe may be caused by extensive microperfusion contamination of the ADC and this does not affect the molecular diffusion obtained by IVIM [22]. This effect of microperfusion contamination in the left lobe is also supported by the observed tendency of increased D_fast_ in segment II against the trend in all other segments.

The study design might have yielded additional insights if pharmacological approaches existed to increase hepatic diffusion and/or perfusion. This would allow to investigate the causality between the hepatic congestion and microperfusion effects, and secondly the fibrosis grade and hepatic diffusion. Unfortunately we are not aware of any of such pharmacological approaches. Also, the disturbed hepatic perfusion in Fontan patients finds its main cause in the reduced cardiac output, which cannot be easily manipulated in terms of study design.

## Conclusions

The decreasing hepatic ADCs in patients with a Fontan circulation reflect decreases in hepatic microperfusion rather than any change in molecular diffusion. However, with the time elapsed since the Fontan operation molecular diffusion and ADC decreased while microperfusion remained stable. This indicates that after Fontan operation initial blood flow effects on the liver are followed by intracellular changes preceding the formation of fibrosis and cirrhosis.

The current study is the first to show with IVIM-DWI that, in a Fontan circulation, the development of liver fibrosis or cirrhosis varies between the different liver segments, potentially depending on the degree of arterial blood supply.

## Supporting information

S1 Appendix The Fontan operation. (DOCX)
