# Supplementary material for: Diminished liver microperfusion in Fontan patients: A biexponential DWI study
Source: PLoS One. 2017 Mar 3;12(3):e0173149. doi: 10.1371/journal.pone.0173149 (PMC5336266; doi:10.1371/journal.pone.0173149)
Supplement: S1 Appendix — (DOCX) [file pone.0173149.s001.docx]

**S1 Appendix. The Fontan operation**

The Fontan operation is currently the treatment-of-choice for patients who are born with a univentricular heart which is not suitable for a biventricular repair [1,2]. With the Fontan operation, the right atrium or both caval veins are surgically connected to the pulmonary artery, thereby bypassing the subpulmonary ventricle. This means that the systemic venous return flows passively through the pulmonary vascular bed, without the aid of a pumping ventricle. As a consequence, Fontan patients suffer from chronically elevated systemic venous pressure and decreased cardiac output due to decreased ventricular preload and increased ventricular afterload. Over four decades, the short term survival after the Fontan operation improved significantly [3]. However, patients who underwent a Fontan operation are prone to develop several complications on the long-term. The liver is one of the organs that suffer from the unphysiologic circumstances. Both the increased systemic venous pressure and the decreased cardiac output are thought to be underlying causes of the progressive liver damage in the Fontan circulation. The liver damage in the Fontan circulation was first recognized in a 15-year-old girl with severe systemic hypertension due to a conduit stenosis [4]. Nowadays, more evidence is emerging that liver damage is not restricted to single patients with adverse hemodynamic complications, but is inherently related to the un-physiological circumstances of the Fontan circulation [5]. Liver damage in the Fontan circulation presents with disturbed transaminases, coagulation disorders, and can eventually lead to liver fibrosis-, cirrhosis and even hepatocellular carcinoma [6-8]. Because a liver biopsy (which is considered the golden standard) is hazardous in Fontan patients, the search for alternative measures to assess liver fibrosis and cirrhosis is ongoing.

**References**

1. Fontan F, Baudet E. Surgical repair of tricuspid atresia. Thorax. 1971;26: 240-248.

2. Kreutzer G, Galindez E, Bono H, De Palma C, Laura JP. An operation for the correction of tricuspid atresia. J Thorac Cardiovasc Surg. 1973;66: 613-621.

3. Wolff D, van Melle JP, Ebels T, Hillege H, van Slooten YJ, Berger RM. Trends in mortality (1975-2011) after one- and two-stage Fontan surgery, including bidirectional Glenn through Fontan completion. Eur J Cardiothorac Surg. 2014;45: 602-609.

4. Lemmer JH, Coran AG, Behrendt DM, Heidelberger KP, Stern AM. Liver fibrosis (cardiac cirrhosis) five years after modified Fontan operation for tricuspid atresia. J Thorac Cardiovasc Surg. 1983;86: 757-760.

5. Wolff D, van Melle JP, Dijkstra H, Bartelds B, Willems TP, Hillege H, et al. The Fontan circulation and the liver: A magnetic resonance diffusion-weighted imaging study. Int J Cardiol. 2016;202: 595-600.

6. Johnson JA, Cetta F, Graham RP, Smyrk TC, Driscoll DJ, Phillips SD, et al. Identifying predictors of hepatic disease in patients after the Fontan operation: a postmortem analysis. J Thorac Cardiovasc Surg. 2013;146: 140-145.

7. Asrani SK, Asrani NS, Freese DK, Phillips SD, Warnes CA, Heimbach J, et al. Congenital heart disease and the liver. Hepatology. 2012;56: 1160-1169.

8. Asrani SK, Warnes CA, Kamath PS. Hepatocellular carcinoma after the Fontan procedure. N Engl J Med. 2013;368: 1756-1757.
